# A brief review of the role of training in near-tool effects

**DOI:** 10.3389/fpsyg.2013.00576

**Published:** 2013-09-03

**Authors:** Liana E. Brown, Melvyn A. Goodale

**Affiliations:** ^1^Department of Psychology, Trent UniversityPeterborough, ON, Canada; ^2^Departments of Psychology, Physiology and Pharmacology, The Brain and Mind Institute, The University of Western OntarioLondon, ON, Canada

**Keywords:** peripersonal space, motor learning, motor control, multisensory integration, tools

## Abstract

Research suggests that, like near-hand effects, visual targets appearing near the tip of a hand-held real or virtual tool are treated differently than other targets. This paper reviews neurological and behavioral evidence relevant to near-tool effects and describes how the effect varies with the functional properties of the tool and the knowledge of the participant. In particular, the paper proposes that motor knowledge plays a key role in the appearance of near-tool effects.

One of the most surprising and interesting findings in research on near-hand effects is the possibility that they may also apply to the functional end of hand-held tools and virtual tools. Perhaps one of the reasons tool-users behave as if the tool is a part of their body is that the same neural mechanism responsible for signaling the visual and tactile border-zone between our bodies and surrounding space adapts to code the space surrounding tools. So when an experienced hockey player carries the puck up the ice on his stick, it is possible that his brain responds to visual and tactile information about the puck as if he were carrying it in his hands.

The space within reach, known as peripersonal space, is represented by visual-tactile bimodal neurons. Bimodal neurons, discovered in the monkey ventral premotor cortex (PMv), putamen, and the intraparietal sulcus, have overlapping visual and tactile receptive fields (vRFs and tRFs, respectively), typically on the face or hand (Rizzolatti et al., 1981a,b; Graziano and Gross, 1993; Graziano et al., 1994; Fogassi et al., 1996; Graziano, 1999; Graziano and Gandhi, 2000). Some also integrate auditory information (Graziano, 1999; Schlack et al., 2005). These neurons receive proprioceptive information (Rizzolatti et al., 1981a,b; Graziano et al., 1994; Graziano, 1999) and their vRFs remain anchored to the hand when it moves to a new location (Graziano et al., 1994; Graziano, 1999). Space near the hands (and face) is represented more densely than space far from the hands and face (Graziano and Cooke, 2006), and bimodal-cell firing rates gradually decay as the distance between the stimulus and the edge of the tactile RF increases (Graziano et al., 1994; Graziano, 1999). Interestingly, the vRFs of these neurons surround and extend beyond the tRF such that visual stimuli appearing near but not touching the skin (within the vRF alone) can also recruit these neurons. In short, visual information presented near the hands may recruit bimodal neurons, whereas visual information presented away from the hands may not. This recruitment has been cited as a possible mechanism underlying the special properties of peripersonal space and may play an important role in the representation of our body schema (Cardinali et al., 2009a).

Research suggests that when a monkey uses a hand-held tool, the size of the bimodal-cell vRF adapts to encompass the tool and that tool-use training plays a key role in this adaptation. Iriki et al. (1996) recorded from visual-tactile bimodal cells in the anterior bank of the intraparietal sulcus (a-IPS) both before and after monkeys practiced using a light, plastic rake to retrieve a food pellet. They recorded activity in both “distal” cells, whose tRF was on the skin of the hand, and “proximal” cells, whose tRF was on the skin of the shoulder. Before training, the vRF of distal cells surrounded the skin and space near the hands only, but after 5 min of rake use, the same neurons responded to stimuli presented near the tool tip. The vRF of proximal cells showed a similar pattern of adaptation: before training, the vRFs encompassed reach space of the arm and hand, but after training, the same vRFs grew to encompass the area reachable with the tool-in-hand. Importantly, these changes were induced only after active tool use, and not after the monkeys held the tool passively for the same duration. The importance of training was underscored by Obayashi et al. (2000), who reported that hand-movement training caused previously unimodal somatosensory neurons in the post-central gyrus of the macaque parietal cortex (Iwamura et al., 1993) to become sensitive to near-hand visual stimuli (i.e., unimodal tactile neurons became bimodal neurons after training). In short, active use of the hand (Obayashi et al., 2000) or a hand-held tool (Iriki et al., 1996; Maravita and Iriki, 2004) may change how the CNS represents the space surrounding the hand or tool.

## Near-tool effects in humans are dependent on activity

As documented in this issue, there has been a great deal of research exploring the possibility that humans also possess neural systems that respond when visual stimuli are presented near the hand (Makin et al., 2007; Brozzoli et al., 2011; Gentile et al., 2011) and, in parallel, whether presenting a visual target near a tool will also influence the speed, accuracy, and variability of perception. One indicator of near-tool effects on perception in humans is the increased efficiency with which targets presented near the tool are processed (Maravita et al., 2001; Farnè et al., 2005a,b, 2007; Holmes et al., 2007a, 2008; Kao and Goodale, 2009; Short and Ward, 2009; Reed et al., 2010; Brown et al., 2011; Gozli and Brown, 2011; but see Holmes et al., 2007b). Another set of studies has shown that the features of visual processing that distinguish peripersonal (near) from extrapersonal (far) space are reduced or eliminated when patients and/or healthy participants use a tool that extends reach (Berti and Frassinetti, 2000; Pegna et al., 2001; Witt et al., 2005; Longo and Lourenco, 2006; Gamberini et al., 2008; Osiurak et al., 2012; Seraglia et al., 2012; Witt, 2011; but see de Grave et al., 2011).

The importance of active tool use (vs. passive holding) has been demonstrated in a series of studies study conducted by Farnè and his colleagues (Farnè and Làdavas, 2000; Farnè et al., 2005a,b, 2007) on visual tactile extinction. This cross-modal version of tactile extinction is observed in some patients with unilateral lesions (typically involving the parietal cortex). Even though a patient might be able to feel a tactile stimulus when it is presented by itself on the hand contralateral to the lesion, he or she will often fail to report that same stimulus when a visual stimulus is presented simultaneously near the ipsilesional hand; i.e., detection of the tactile stimulus is extinguished. In a case study of visual-tactile extinction, Farnè et al. (2005a) showed that extinction of a tactile stimulus presented on the contralesional hand also occurred when a visual stimulus was presented near the tip of a tool held in the ipsilesional hand after the patient had used the tool to rake in objects for 5 min, but not after the patient spent that time passively holding the tool. In a follow-up study, Farnè et al. (2005b) demonstrated that the strength of cross-modal extinction by the presentation of visual stimuli at the tip of the tool depended on the length of the tool used during training. Patients who trained with a 60-cm tool showed greater cross-modal extinction when holding a 60-cm tool than a 30-cm tool, and patients who trained with a 30-cm tool showed greater cross-modal extinction when holding the 30-cm tool. Finally, patients who held a hybrid tool in their ipsilesional hand that was 60-cm in length, but whose functional rake component was placed 30 cm from the hand, showed greater cross-modal extinction of the tactile stimulus on the contralesional hand when the visual stimulus was placed at 30 cm than at 60 cm. These results indicate that the functional length of the tool is more important than the physical length, and suggest that active training allows the user to learn about the capabilities of the tool from multiple sensory modalities.

Findings by Kao and Goodale (2009) also showed that near-tool effects are dependent on training and specific to the tool used during training. In their study, healthy participants trained with either a fake hand or a small rake. After training, fake-hand trainees showed enhanced detection for targets presented on the fake hand but not the rake, and rake-trainees showed enhanced detection for targets presented on the rake but not the fake hand. Enhanced target detection was documented only when the target was presented on the surface of the tool viewed during training. Reed et al. (2010) also found enhanced detection of targets presented near the tip of a rake that had been used to manipulate sand in a Zen garden. Reed et al. found that detection benefits were restricted to one side of the tool, but in this study, benefits were restricted to the functional side of the tool (tine-side). Together with the study by Farnè et al. (2005b), these studies suggest that near-tool effects do not always generalize to the entire tool, and that knowledge of the tool's recent function plays a key role in the appearance of near-tool effects.

Although many studies have focused on visual adaptation, tool-use-dependent changes in the representation of space around the tool have also been documented in the auditory modality. Serino et al. (2007) found that when sighted participants were tested immediately after taking a cane in-hand, they responded more quickly to sounds presented near the hand than near the cane tip. After one day of cane use, this difference disappeared, indicating tool-use-related spatial adaptation. Experienced blind cane users, by contrast, did not need additional training to exhibit spatial adaptation to the tool. These results suggest that tool-related spatial adaptation is applicable to a broad range of sensory modalities and they highlight the importance of tool-use experience.

## Near-tool effects depend on motor control knowledge

The observation that near-tool effects depend on some training suggests that there is a process of learning or recall of tool-related knowledge that needs to be invoked to induce spatial adaptation near the tool. One possibility is that this knowledge is motor in nature. Arbib et al. (2009) argued that tool representation must include both a mapping of tool reach to spatial locations so that peripersonal space can adapt to accommodate reach, and a mapping of tool function to hand movements so that the function of the tool can be linked to the hand movements that are required to effect that function (see also Frey, 2007). Both Làdavas and Serino (2008) and Makin et al. (2012) note that neural systems responsible for the sensory representation of the body overlap substantially with regions involved in sensorimotor control and emphasize the distinct visuomotor processing advantages that these links may provide.

The acquisition of motor knowledge involves establishing a reliable predictive relationship between the planned motor command and the visual, proprioceptive, and dynamic tactile sensory consequences resulting from its execution (Wolpert, 1997; Wolpert and Kawato, 1998; Flanagan and Beltzner, 2000; Flanagan et al., 2003). This acquired model of the limb (an internal model) generates motor commands—the directive for muscle activations—from planned movement trajectories. A second, forward model of the limb system predicts the resultant sensory outcomes from the motor command. These models must account for many factors, including physical factors like the mass and lengths of limb segments, gravity, and both directly- and indirectly-generated (interaction) torques about the joints (Sainburg et al., 1995, 1999; Wolpert and Kawato, 1998; Gribble and Ostry, 1999). Tool motor learning involves acquiring the ability to predict the sensory information that will result from the combination of limb and tool movement. When tools are added to the limb or hand, both the forward and inverse model must adapt to account for the additional mass and torque applied to the limb system (Sainburg et al., 1999; Bagesteiro and Sainburg, 2003; Wang and Sainburg, 2004). If participants have worked with the tool before, this adaptation may be expedited as they may access stored information about the tool's inertial profile (Haruno et al., 2001).

In short, motor knowledge may play a role in near-tool effects because it allows the user to make predictions about the spatial location of the working end of the tool as it is moved, linking limb, hand, and tool posture (signaled by the somatosensory system) to locations in space beyond the body (usually signaled by the visual system). Put differently, we may need to be able to control and reliably predict the tool's actions before changes in how space around the tool is represented can be implemented (e.g., adaptation of the vRF of visual-tactile bimodal neurons). Although not explicitly linked to motor knowledge *per se*, a promising computational model of the effects of tool use on the representation of peripersonal space relies on a predictive mechanism that contributes to spatial adaptation in multisensory cells (Magosso et al., 2010).

This view is supported by work showing that near-tool effects can be directly linked to action preparation (Collins et al., 2008; Brozzoli et al., 2010) and by studies indicating that active tool training changes participants' implicit representation of the extent and shape of their own limb (Cardinali et al., 2009a,b, 2012; Sposito et al., 2012; Canzonieri et al., 2013). Berti and Frassinetti (2000) showed that near-space hemispatial neglect expanded to far space when neglect patient PP held a stick-pointer but not when she held a laser-pointer in which the sensation of the inertial forces and changes in the location of the laser light are uncoupled. A similar distinction has been demonstrated in healthy participants (Gamberini et al., 2008; Witt, 2011; but see Davoli et al., 2011). This latter result suggests that there may be a special role for objects whose reach (length) can be both seen and felt via tactile and proprioceptive cues signaling their inertia (Carello et al., 1998; Carello and Turvey, 2004).

A strong prediction of the motor knowledge hypothesis is that when participants are given a tool that is unfamiliar in terms of either inertial, spatial, or temporal dynamics, they will not show enhanced processing near that tool until its dynamics can be predicted in a way that the sensorimotor system finds useful. Brown et al. (2011) tested this hypothesis by presenting participants with a tool that had an unseen, off-center mass load, a feature that controlled for participants' tool-experience history. Testing revealed that participants who trained actively with the tool could control the tool better than people who received passive or no training, and that only participants in the active training group responded more quickly to detection targets when the tool tip was placed near rather than far from the display. The results support previous findings showing that active tool use plays a role in near-tool effects, and they suggest that active tool use is important for learning about the inertial dynamics of the tool.

The proposal that motor knowledge plays a key role in near-tool effects can also account for studies showing that near-tool effects can be detected with little or no training. For example, Holmes et al. (2007a) reported a reduction in interference associated with near-tool visual stimuli after only a very short duration of active tool use, and studies have found cross-modal interference for near-tool visual stimuli after simple real (Maravita et al., 2002) and virtual (Sengül et al., 2012) tool holding. Both children and adults adapted their estimates of reach distance to a pointing tool after a brief exposure (Caçola and Gabbard, 2012; Osiurak et al., 2012; Caçola et al., 2013), although older children (11+) adapted more effectively than younger children, and the efficiency of adaptation improves with age. It is possible that these studies found rapid adaptation of peripersonal space with tool use because, without exception, they used simple tools and/or toys (sticks, pointers, or toy rakes). Participants, even children, may have been able to (1) learn to control the tool very quickly, or (2) take advantage of their prior experience with tools to recall the necessary motor control information quickly (Imamizu et al., 2007; Serino et al., 2007; Massen and Prinz, 2009).

There has also been a great deal of interest in the role that multisensory systems and the motor system may be playing in our ability to treat virtual tools as if they are extensions of ourselves. In this domain, there has been emphasis on the idea that mechanisms involved in defining peripersonal space can extend to virtual tools (e.g., Bassolino et al., 2010; Sengül et al., 2012) and that motor agency may play a key role in this extension (Longo and Haggard, 2009; Short and Ward, 2009). Agency, in this case, is defined as the understanding that one's actions consistently cause the movement of a remotely displayed item or tool (e.g., mouse cursor). Agency has been manipulated by introducing temporal delays between movements made by the actor and movements of the virtual tool (Shimada et al., 2005; Longo and Haggard, 2009) or by presenting synchronous visual feedback only intermittently (Short and Ward, 2009; Nahab et al., 2011). These manipulations have shown that when one interferes with perceived agency for the observed limb, the visual facilitation associated with presenting stimuli near the virtual tool diminishes (Shimada et al., 2005; Longo and Haggard, 2009; Short and Ward, 2009; Nahab et al., 2011). Gozli and Brown (2011) investigated the role that motor control plays in near-virtual-tool effects by manipulating the spatial mapping between the movements of the user and the observed motion of the virtual tool. Participants were briefly exposed to three different spatial mappings between hand movements and motion of an on-screen mouse cursor. This mapping was either familiar (standard hand-cursor mapping), unfamiliar (reversed mapping), or absent (movements of mouse produced no cursor motion). Participants' ability to quickly detect the onset of cursor motion was then tested and revealed that participants exposed to the familiar-mapping condition responded more quickly than those exposed to the unfamiliar or no-mapping conditions. Given that participants in the unfamiliar mapping condition could still cause movements of the cursor in a temporally-consistent manner (they still had agency) but could not control them (their movements were slow and deliberate), Gozli and Brown argued that near-tool effects depend more on their knowledge of tool motor-spatial control than on tool agency.

Together, these results indicate that motor knowledge about the inertial and spatial dynamics of a tool play an important role in near-tool effects. When people are presented with standard tools, like wooden pointers, or with light easy-to-manipulate tools, like toy rakes, they are able to acquire or recall motor control information quickly. When the system is challenged by presenting participants with tools with novel inertial dynamics or with unusual spatial mappings, the dependence of near-tool effects on motor knowledge is more easily revealed. We may need to be able to control and reliably predict the tool's actions before changes in which the space around the tool is represented can be implemented in any sensory modality. This principle suggests that the future application of near-tool effects in occupational, educational, or rehabilitation settings will require close attention to the role of motor knowledge in tool use—and by extension its role in promoting the detection of stimuli around the “business-end” of tools.

### Conflict of interest statement

The authors declare that the research was conducted in the absence of any commercial or financial relationships that could be construed as a potential conflict of interest.
